# Psychobiotics and the microbiota–gut–brain axis: Emerging paradigms in mental health modulation

**DOI:** 10.1113/EP093301

**Published:** 2026-02-28

**Authors:** Amir Arsalan Ghahari, Mehrdad Nourizadeh, Mehrdad SalekShahabi, Shaghayegh Davari, Saeid Mohammadzadeh Mounesyar

**Affiliations:** ^1^ Neurosciences Research Center Tabriz University of Medical Sciences Tabriz Iran; ^2^ Department of Medicine, TaMS.C. Islamic Azad University Tabriz Iran

**Keywords:** anxiety and depression, brain‐derived neurotrophic factor (BDNF), *Lactobacillus* and *Bifidobacterium*, microbiota–gut–brain axis, neuroinflammation, psychobiotics

## Abstract

The global rise in mental health conditions has prompted interest in interventions that act beyond conventional psychopharmacology. Psychobiotics, broadly understood as live microorganisms or microbe‐derived products that interact with the microbiota–gut–brain axis, have been suggested to exert neuroactive effects through neural, immune, endocrine and metabolic routes. This narrative review synthesizes recent preclinical, mechanistic and early clinical observations. Experimental studies show that selected strains can modulate cytokine signalling, influence stress‐responsive systems such as the hypothalamic–pituitary–adrenal axis, and support synaptic plasticity via factors such as brain‐derived neurotrophic factor. A limited number of human trials using well‐characterized *Lactobacillus* and *Bifidobacterium* strains have reported improvements in affective and stress‐related outcomes, but these effects are generally small to moderate, more apparent in adjunctive than stand‐alone use, and dependent on strain, dose, population and intervention length (typically 4–12 weeks). Evidence on neurodevelopmental conditions (e.g., autism spectrum disorder, attention‐deficit/hyperactivity disorder) remains preliminary, based on small and heterogeneous samples. Across studies, key constraints include methodological heterogeneity, incomplete strain‐level reporting, and gaps in mechanistic resolution that make it difficult to link microbial shifts to psychiatric benefit. Emerging microbiome‐ and metabolomics‐informed approaches may help identify likely responders and improve translational precision, but they are not yet ready for routine clinical application. Overall, psychobiotics should currently be viewed as a promising adjunct within integrative mental health care, warranting larger, standardized trials with clearly defined strains, doses and mechanistic endpoints.

## Abstract (highlights)

- **What is the topic of this review?**
  This review examines the emerging field of psychobiotics, focusing on how specific microorganisms and their metabolites influence mental health through the microbiota–gut–brain axis.
- **What advances does it highlight?**
  Recent mechanistic insights link psychobiotics to neuroimmune modulation, hypothalamic–pituitary–adrenal axis regulation, neurotransmitter balance and neuroplasticity. The review emphasizes evidence from clinical trials showing strain‐specific benefits in mood and anxiety disorders, as well as early findings in neurodevelopmental and severe mental illness. It also outlines precision approaches, including microbiome profiling and biomarker‐guided interventions, that may enhance translation into psychiatric practice.

## 1 INTRODUCTION

Mental and neurodevelopmental disorders, including autism spectrum disorder (ASD), attention‐deficit/hyperactivity disorder (ADHD), schizophrenia, anxiety disorders, post‐traumatic stress disorder (PTSD) and depression, impose a major global burden (GBD 2019 Mental Disorders Collaborators, 2022). The World Health Organization (WHO) estimates that mental illness is among the leading causes of disability‐adjusted life years (DALYs), affecting more than 970 million people worldwide. Although pharmacological options have expanded, widely used treatments such as atypical antipsychotics, benzodiazepines, selective serotonin reuptake inhibitors and mood stabilizers are still constrained by delayed onset, partial or non‐response, adverse effects and poor adherence, particularly in treatment‐resistant populations (Accettulli et al., 2022). These limitations have increased interest in integrative approaches that target biological systems beyond canonical monoaminergic pathways.

The microbiota–gut–brain axis (MGBA) is a bidirectional network connecting the gastrointestinal (GI) tract with immune and endocrine signalling and the central nervous system (CNS). Growing evidence suggests that the gut microbiome and its metabolites can influence cognition, emotion regulation, synaptic signalling and neurodevelopment (Wu et al., 2025). Dysbiosis, defined as disruption of gut microbial communities, has been linked to multiple psychiatric and neurological conditions, including depression, ASD, schizophrenia, Parkinson's disease and generalized anxiety disorder (Generoso et al., 2020), encouraging investigation of microbiome‐targeted strategies in psychiatric research and care (Moerkl et al., 2020).

Psychobiotics were initially defined by Dinan et al. as live organisms or microbe‐derived substances that, when ingested in adequate amounts, confer mental health benefits via the MGBA (Dinan et al., 2013). In contrast to conventional probiotics that primarily target GI health, psychobiotics are framed as microbiome‐based agents that may act through neuroactive pathways, including neurotransmitter‐related signalling (e.g., GABA, serotonin), immunomodulation, hypothalamic–pituitary–adrenal (HPA) axis regulation, and support of neuroplasticity (Del Toro‐Barbosa et al., 2020). The concept has broadened to include not only live strains but also postbiotics, synbiotics, para‐psychobiotics and prebiotics, expanding the therapeutic and safety spectrum, particularly for immunocompromised or vulnerable populations (Zielińska et al., 2022). An explanation of the terms used is provide in Box 1.

**Box 1. Working definitions used in this review**

- **Probiotics**: live microorganisms that, when administered in adequate amounts, confer a health benefit on the host.
- **Psychobiotics**: probiotics or microbe‐derived interventions that improve mental health or stress‐related outcomes through MGBA pathways such as vagal signalling, immune modulation, HPA‐axis regulation, or metabolite‐driven neuroplasticity.
- **Prebiotics**: non‐digestible substrates (e.g., fructooligosaccharides, galactooligosaccharides) that selectively support beneficial gut microbes and may enhance psychobiotic effects by enriching short‐chain fatty acid (SCFA)‐producing taxa.
- **Synbiotics**: combinations of a defined probiotic strain with a compatible prebiotic substrate to improve colonization, metabolite output and stability.
- **Postbiotics**: non‐viable microbial products or metabolites (including SCFAs, tryptophan derivatives, and cell‐wall components) that can exert immunomodulatory or neuroactive effects without requiring live colonization.
- **Para‐psychobiotics**: inactivated but structurally intact microbial preparations that retain bioactivity at the gut–immune interface and have been reported to improve outcomes such as sleep or anxiety in stressed individuals.

Preclinical studies indicate that selected preparations can modulate synaptic plasticity, neuroinflammation, stress‐hormone responses and behaviour, with some effects depending on vagus nerve integrity (Andreeva et al., 2022; Jia et al., 2024). Early randomized controlled trials (RCTs) in humans report improvements in perceived stress, anxiety, sleep quality, depressive symptoms, and selected outcomes in ASD and ADHD, but effects are typically modest and heterogeneous across strains, doses, populations and follow‐up periods (Magalhães‐Guedes et al., 2020). Translation is further limited by short trial durations (often 4–12 weeks), small and diverse samples, inconsistent strain‐level reporting, and host‐dependent variability related to diet, genetics, concomitant medications (especially antibiotics and psychotropics) and baseline microbiota composition (Mosquera et al., 2024). Regulatory ambiguity and limited standardization in labelling and classification of microbiome‐based therapeutics also slow clinical adoption (Andreeva et al., 2022).

With an emphasis on neurobiological mechanisms, strain‐specific profiles, clinical applications and translational challenges, this narrative review evaluates current evidence and clinical promise in psychobiotic research. We synthesized findings across molecular biology, psychiatry and microbiome science, based on searches of PubMed, Scopus, Web of Science and Google Scholar for studies published between 2010 and 2025 using terms such as ‘psychobiotics’, ‘microbiota–gut–brain axis’, ‘probiotics’, ‘anxiety’, ‘depression’, ‘ASD’, ‘ADHD’, ‘neuroinflammation’, and ‘BDNF’. We prioritized original preclinical, clinical and mechanistic studies that assessed neuroimmune, neuroendocrine, or behavioural outcomes; used review papers selectively for background; and excluded studies with unclear strain identification, limited mechanistic detail, or a primary focus outside psychiatry, to balance depth with translational relevance and maintain transparency in study selection.

## 2 THE MICROBIOTA–GUT–BRAIN AXIS: NEUROIMMUNE AND NEUROENDOCRINE INTERFACES

**FIGURE 1:**
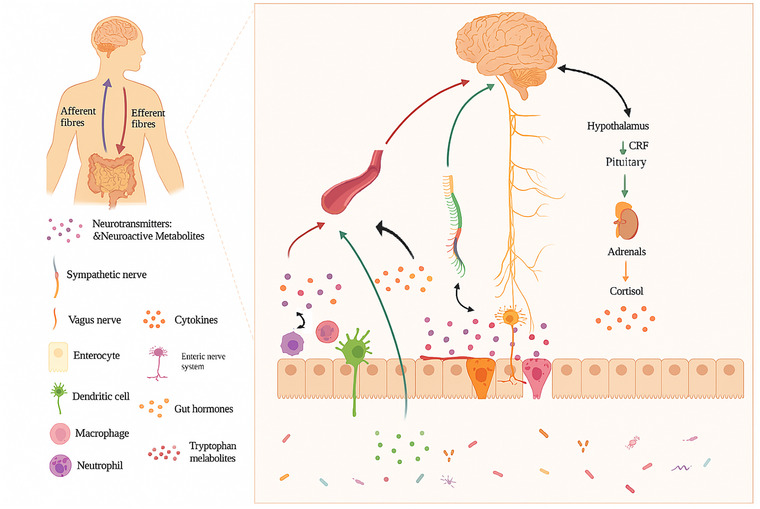
Gut microbiota–brain communication occurs through parallel pathways involving microbial metabolites (e.g., neurotransmitters and SCFAs) that can influence the CNS, vagus nerve signalling from the gut to the brain, and circulating mediators (hormones and immune‐derived cytokines) that affect brain function after entering systemic circulation. BBB, blood–brain barrier; SCFAs, short‐chain fatty acids; VN, vagus nerve.

The MGBA is a bidirectional communication system linking the GI microbiota with the CNS. Psychobiotics exert their neuroactive and therapeutic effects through four interrelated routes that together provide the biological background for all subsequent sections (Del Toro‐Barbosa et al., 2020; Garzone et al., 2025) (Figure 1).

### 2.1 Neural pathway: Vagus nerve and enteric nervous system

Neural signalling is primarily conveyed through the vagus nerve and the enteric nervous system (ENS). Afferent vagal fibres project from the gut to emotion‐ and memory‐related regions such as the amygdala and hippocampus. In a preclinical, vagus‐dependent model, *Lactobacillus rhamnosus* JB‐1 altered GABA receptor expression and reduced anxiety‐like behaviour. Subdiaphragmatic vagotomy abolished this effect, confirming that an intact vagus is necessary for this psychobiotic action (Bravo et al., 2011; Neska et al., 2024). This strain is a clear example of a neural and vagal route.

### 2.2 Immune pathway: Gut barrier, lipopolysaccharide and cytokines

Gut microbes maintain systemic immune tone by supporting epithelial integrity and modulating cytokine secretion (Mazziotta et al., 2023). Dysbiosis can increase intestinal permeability and facilitate the translocation of pro‐inflammatory molecules such as lipopolysaccharide (LPS). This induces peripheral cytokine release, including interleukin (IL)‐6 and tumour necrosis factor α (TNF‐α), which has been associated with depressive symptoms and cognitive decline (Chen et al., 2022). Preclinical work in an LPS‐induced model of neuroinflammation showed that probiotic intervention ameliorated LPS‐driven injury and was accompanied by favourable changes in Aβ‐related proteins (Aβ1–42, APP, γ‐secretase) and increased brain‐derived neurotrophic factor (BDNF) relevant to fetal neurodevelopment (Kar et al., 2022). Human and animal studies with *Lactobacillus helveticus* and *Bifidobacterium longum* also report decreased pro‐inflammatory cytokines and increased IL‐10, which indicates that these strains act mainly through an immune‐modulatory route (Zakharova et al., 2022).

### 2.3 Endocrine pathway: HPA axis regulation

Stress activates the HPA axis and results in altered cortisol levels, changes in GI motility and mood disturbances (Oroojzadeh et al., 2022). Germ‐free rodents display exaggerated corticosterone responses, which are normalized after microbial colonization. This shows that a normal microbiota is required for correct HPA reactivity (Sharma et al., 2021). In both animal and human studies, *Lactobacillus casei* Shirota and *Clostridium butyricum* have been reported to lower cortisol and reduce stress reactivity, which indicates a microbiota‐sensitive endocrine route (Aziz et al., 2024; Samtiya et al., 2022). In humans, *B. longum* 1714 has also been shown to reduce perceived stress and cortisol and to improve cognition, so this clinically studied strain can be placed in the same HPA and stress regulation pathway (Allen et al., 2016).

### 2.4 Metabolic pathways: Microbial short‐chain fatty acids and neuroplasticity

Commensal bacteria ferment dietary substrates to generate short‐chain fatty acids (SCFAs) such as acetate, propionate and butyrate. These metabolites can enter the circulation, cross the blood–brain barrier (BBB) and affect microglial activation, neuroinflammation and the expression of neurotrophic factors, particularly BDNF, in hippocampal and cortical regions (Gupta et al., 2026). Multiple preclinical studies have reported antidepressant‐like or pro‐cognitive effects that are consistent with SCFA‐driven support of synaptic plasticity and neurogenesis (Roy et al., 2024). Psychobiotic strategies that increase endogenous SCFA‐producing taxa, for example by using prebiotics or synbiotics, therefore act mainly through this metabolic arm (Binda et al., 2024).

### 2.5 Integrative view

**FIGURE 2:**
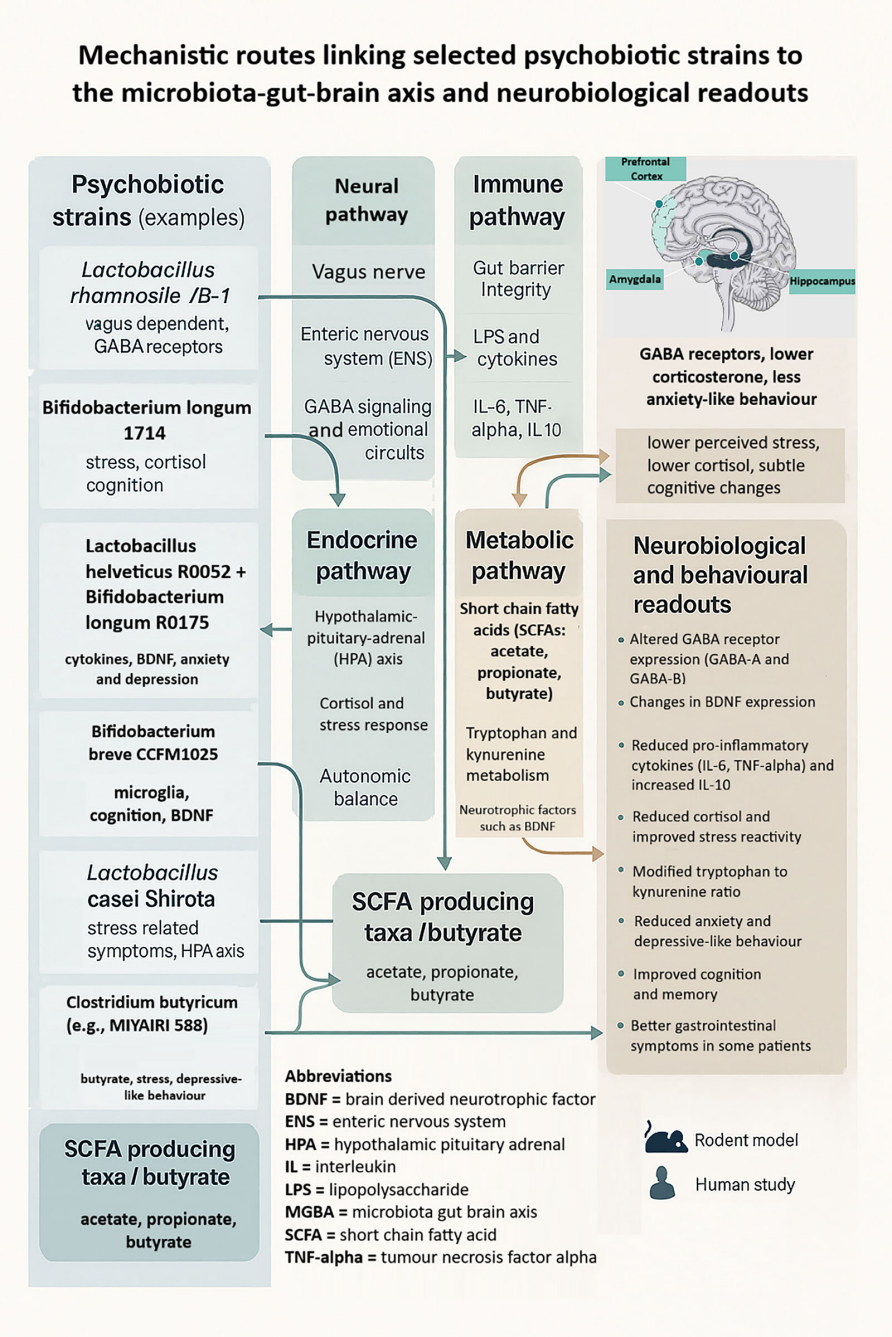
Mechanistic routes linking selected psychobiotic strains to the microbiota–gut–brain axis and neurobiological readouts. The left panel lists exemplar strains that act through neural, immune, endocrine and metabolic pathways, including vagal signalling, cytokine modulation, HPA axis regulation and short chain fatty acid (SCFA) production. These routes converge on limbic and prefrontal brain regions and are associated with changes in GABA receptors, BDNF, inflammatory cytokines, cortisol, and behavioural outcomes such as anxiety, mood, cognition and gastrointestinal symptoms. Icons indicate whether supporting data come from rodent models or human studies. BDNF, brain derived neurotrophic factor; ENS, enteric nervous system; HPA, hypothalamic–pituitary–adrenal; IL, interleukin; LPS, lipopolysaccharide; MGBA, microbiota–gut–brain axis; SCFA, short chain fatty acid; TNF alpha, tumour necrosis factor alpha.

The four routes outlined above explain how the MGBA can influence behaviour, emotion and cognition (Gupta et al., 2026). Unlike many psychiatric drugs that target a single CNS receptor, psychobiotics can rebalance host physiology at several regulatory levels at the same time, which facilitates their use alongside conventional treatments (Dacaya et al., 2025; Sharma et al., 2021) (Table 1, Figure 2).

**TABLE 1:** Mechanisms of psychobiotic action along the MGBA.

Strain/class	Primary pathway(s)	Model (rodent/human)	Biomarker/readout	Key citation
*Lactobacillus rhamnosus* JB‐1	Neural (vagus), GABA receptor modulation	Rodent	↓ anxiety‐like behaviour, altered GABA_A_/GABA_B_ expression	Bravo et al. (2011)
*Bifidobacterium longum* 1714	Endocrine (HPA), stress modulation, cognition	Human	↓ perceived stress, ↓ cortisol, altered brain activity	Allen et al. (2016)
*Lactobacillus helveticus* + *B. longum* (immune active strains)	Immune, gut barrier, cytokine shift	Human/rodent	↓ IL‐6, ↓ TNF‐α, ↑ IL‐10	Mohammadi et al. (2019)
*Clostridium butyricum*/*L. casei* Shirota	HPA normalization, endocrine–GI link	Human/rodent	↓ cortisol, ↓ stress reactivity	Takada et al. (2016)
SCFA‐producing species/postbiotics (butyrate)	Metabolic, SCFA → BDNF, microglia	Rodent	↑ BDNF, ↓ neuroinflammation, antidepressant‐like effects	Yamawaki et al. (2018)
Probiotics in LPS‐induced neuroinflammation	Immune → Aβ processing → BDNF	Rodent, maternal–fetal	↓ LPS injury, changes in Aβ1‐42, APP, γ‐secretase, ↑ BDNF	Kar et al. (2022)

## 3 THE MECHANISTIC VARIETY OF PSYCHOBIOTICS: FROM MICROBIAL METABOLITES TO LIVE STRAINS

Building on the MGBA framework outlined earlier, psychobiotics can be grouped into product classes that differ in viability, safety profile and their predominant route of action. While the original concept emphasized live probiotic strains, current usage also includes prebiotics, synbiotics, postbiotics and inactivated para‐psychobiotics. Across classes, effects ultimately converge on the same MGBA signalling arms (neural, immune, endocrine and metabolic), but the relative weighting of these routes varies by formulation and host context.

### 3.1 Probiotic psychobiotics (live strains)

Probiotics are live microorganisms that confer a health benefit when administered in adequate amounts. Well‐characterized *Lactobacillus* and *Bifidobacterium* strains have been linked to neuroactive effects through pathways that include metabolite production (e.g., SCFAs, serotonin precursors, GABA), modulation of vagal signalling, HPA‐axis reactivity and cytokine balance (Allen et al., 2016; Bravo et al., 2011). A central translational point is that these effects are strain‐specific; findings for *L. rhamnosus* JB‐1 or *B. longum* 1714 cannot be generalized across a genus or species. Accordingly, studies should report the exact strain designation and daily dose/colony‐forming units (CFU) to support reproducibility and interpretation (Wang et al., 2019).

### 3.2 Prebiotics

Prebiotics are non‐digestible substrates (e.g., fructooligosaccharides, galactooligosaccharides) that selectively promote beneficial taxa (Dacaya et al., 2025). Their psychobiotic impact is largely indirect because they enrich endogenous SCFA‐producing communities (e.g., *Faecalibacterium prausnitzii*), thereby supporting neuroplasticity, microglial homeostasis and anti‐inflammatory signalling via the metabolic arm of the MGBA (Ramadan et al., 2025). Because their effects depend on resident microbiota, baseline composition and diet are expected moderators of response.

### 3.3 Synbiotics

Synbiotics combine a defined probiotic strain with a compatible prebiotic substrate. This pairing can enhance colonization, metabolite output and formulation stability, which may explain stronger stress‐ or mood‐related effects in some trials compared with probiotics alone (Aziz et al., 2024). Rather than introducing a distinct biological pathway, synbiotics primarily amplify and prolong MGBA signalling already attributed to their probiotic and prebiotic components.

### 3.4 Postbiotics

Postbiotics are non‐viable microbial products or metabolites, including SCFAs (e.g., butyrate, acetate), tryptophan derivatives, and structural components such as peptidoglycan (Gupta et al., 2026). Because they do not require live colonization, postbiotics may be preferable in vulnerable or immunocompromised populations (Wadan et al., 2025). Mechanistically, butyrate has been linked to increased hippocampal BDNF, improved synaptic plasticity and reduced anxiety‐like behaviour in rodents, consistent with metabolic and neurotrophic MGBA signalling (Ramadan et al., 2025). Microbe‐derived products may also reduce LPS‐related immune activation by supporting gut barrier function and downstream cytokine regulation, aligning with immune‐mediated routes reported in maternal–fetal neuroinflammation models (Kar et al., 2022).

### 3.5 Para‐psychobiotics (inactivated cells)

Para‐psychobiotics are non‐viable but structurally intact microbial preparations that retain bioactive surface features (Lai & Shen, 2023). They may reduce the risk of translocation while still engaging gut and immune pattern‐recognition pathways. For example, heat‐killed *Lactobacillus gasseri* has been associated with improved sleep and reduced anxiety in stressed individuals, despite the absence of live colonization (Sharma et al., 2021). Their dominant effects are typically framed as immune‐centric, with secondary neuroendocrine consequences.

### 3.6 Implications

Together, these categories highlight that ‘psychobiotics’ comprise multiple intervention types that converge on MGBA communication channels while differing in feasibility and safety. To reduce categorical drift and improve translational value, future studies should specify the product class, the exact strain (or defined metabolite/constituent), the administered dose and the most plausible dominant pathway, rather than attributing effects broadly to ‘probiotics’ (Aziz et al., 2024).

## 4 THE EFFECTS OF STRAIN‐SPECIFIC PSYCHOBIOTICS: FUNCTIONAL AND GENOMIC DESCRIPTION

The specificity of the microbial strain has a substantial impact on the therapeutic efficacy of psychobiotics (Wadan et al., 2025). Different strains of the same species can show distinct metabolic outputs, behavioural effects and genomic profiles, which makes precise strain‐level identification and functional characterization essential in both clinical and experimental work (Gupta et al., 2026). One of the best‐characterized strains is *L. rhamnosus* JB‐1, which shows robust anxiolytic and antidepressant‐like effects in mice. Bravo et al. demonstrated that JB‐1 reduces corticosterone in chronically stressed animals and modulates GABA_A_ and GABA_B_ receptor expression in the amygdala and hippocampus; these effects disappear after vagotomy, indicating a vagus‐dependent mechanism (Bravo et al., 2011; Hunjan et al., 2025). Importantly, this neuroactive profile is not shared by all *L. rhamnosus* strains, which highlights the distinctiveness of JB‐1 (Yadav et al., 2022).

*Bifidobacterium longum* 1714 is another well‐studied psychobiotic with effects demonstrated in both rodents and humans. In clinical trials it improved memory‐related performance, reduced cortisol, and lowered daily perceived stress, and functional magnetic resonance imaging studies confirmed altered activity in brain regions involved in emotional processing (Gupta et al., 2026). Additional strains, including *Bifidobacterium breve* CCFM1025 and *L. helveticus* R0052, have shown neurotrophic and anti‐inflammatory actions, such as reduced IL‐6, increased hippocampal BDNF, improved cognition, and attenuated microglial activation in neuroinflammatory models (Gupta et al., 2026). Psychobiotic effects may also be enhanced in multispecies formulations. For example, the combination of *L. helveticus* R0052 and *B. longum* R0175 has been reported to alleviate depressive and anxiety symptoms in humans, probably through complementary actions on HPA‐axis activity, neurotransmission and inflammatory cytokines (Kim et al., 2023).

Whole‐genome sequencing, metabolomics, and neurophenotyping are now being used to define composite ‘psychobiotic signatures’ that include gene clusters for short‐chain fatty acid synthesis, GABA production and tryptophan metabolism (Li et al., 2024). These signatures may eventually support predictive selection of strains based on desired immunological or neurochemical outcomes. Overall, strain specificity is a central determinant of psychobiotic efficacy rather than a minor detail. Developing reliable and personalized microbial interventions for mental health will require rigorous validation of functional properties at the strain level in addition to genus and species classification (Yousuf et al., 2025).

## 5 CLINICAL AND PRECLINICAL PERSPECTIVES ON PSYCHOBIOTICS IN MOOD AND ANXIETY DISORDERS

Mood and anxiety disorders are the most frequently explored indications for psychobiotic interventions, partly because they overlap mechanistically with low‐grade inflammation, gut dysbiosis and dysregulation of the HPA axis (Tiwari & Paramanik, 2025).

### 5.1 Preclinical evidence

Rodent studies show relatively consistent signals for a small group of strains. *L. rhamnosus* JB‐1 reduced anxiety‐like behaviour and was associated with vagus‐dependent modulation of GABA receptors in the amygdala and hippocampus (Lachmansingh et al., 2024), which is in line with the neural route outlined earlier. *B. breve* CCFM1025 improved cognition and reduced microglial activation in neuroinflammatory models (Oroojzadeh et al., 2022). These behavioural effects typically co‐occurred with lower circulating IL‐6 and TNF‐α and higher hippocampal BDNF, which supports an anti‐inflammatory and pro‐neuroplastic profile (Zubareva & Melik‐Kasumov, 2021). Because these outcomes have been demonstrated only for specific strains under defined experimental conditions, they should not be generalized to all *Lactobacillus* or *Bifidobacterium* species.

### 5.2 Clinical trials in depression, anxiety and related psychiatric conditions

Randomized trials in major depressive disorder (MDD) and stress‐related states suggest that psychobiotic effects are strain‐ and context‐dependent, and are often more apparent in adjunctive or biologically dysregulated populations. In an 8‐week double‐blind RCT (*n* = 40), Akkasheh et al. reported greater reductions in Beck Depression Inventory (BDI) scores with a multistrain probiotic (*Lactobacillus acidophilus*, *L. casei*, *Bifidobacterium bifidum*; ∼2 × 10^9^ CFU/strain/day) versus placebo, alongside improvements in metabolic and inflammatory markers (Akkasheh et al., 2016). In a three‐arm 8‐week RCT (*n* = 110), Kazemi et al. found that *L. helveticus* plus *B. longum* reduced depressive symptoms more than both a galactooligosaccharide (GOS) prebiotic and placebo, with modest shifts in tryptophan–kynurenine‐related indices (Kazemi et al., 2019).

Adjunctive studies indicate potential additive benefits in more severe cases, although design limitations remain. An 8‐week open‐label controlled inpatient study (*n* = 40) suggested that adding *C. butyricum* MIYAIRI 588 to antidepressants improved depression and anxiety scales more than antidepressants alone, but the absence of blinding limits inference (Miyaoka et al., 2018). In an 8‐week double‐blind adjunctive RCT in selective serotonin reuptake inhibitor (SSRI)‐treated MDD (*n* = 79), *Lactobacillus plantarum* 299v improved attention and verbal learning and altered kynurenine markers, while mood outcomes improved similarly in both arms, indicating primarily cognitive/biochemical effects rather than robust incremental antidepressant efficacy (Rudzki et al., 2019).

In healthy volunteers, *B. longum* 1714 reduced stress‐induced cortisol output and perceived stress and produced small neurocognitive/EEG changes after 4 weeks, consistent with stress‐modulating rather than ‘antidepressant’ effects in low‐symptom samples (Allen et al., 2016). Beyond mood and anxiety, evidence is mixed: in schizophrenia, a 14‐week double‐blind RCT (*n* = 65) found no improvement in Positive and Negative Syndrome Scale (PANSS) scores but reduced severe constipation risk in antipsychotic‐treated patients (Dickerson et al., 2014). In ASD in a small double‐blind crossover trial (*n* = 15), *L. plantarum* WCFS1 altered GI/microbiota measures with unclear behavioural benefit (Parracho et al., 2010), whereas an open‐label microbiota transfer therapy protocol (*n* = 18) reported sustained GI and autism‐scale improvements, though results remain exploratory due to the non‐randomized, unblinded, multi‐component design (Kang et al., 2017).

Overall, these trials suggest that baseline dysregulation (e.g., clinically significant depression, chronic stress or GI disturbance) and intervention parameters (strain identity, dose, duration) shape whether benefits are primarily emotional, cognitive or somatic. Key study parameters are summarized in Table 2.

**TABLE 2:** Key psychobiotic and microbiota‐targeted clinical trials.

Study (population)	Design/*n*/duration	Intervention	Daily dose	Key outcomes & significance
Akkasheh et al. (2016) (MDD)	DB RCT; *n* = 40; 8 weeks	Multistrain probiotic (*L. acidophilus*, *L. casei*, *B. bifidum*)	∼2 × 10^9^ CFU of each strain/day	Larger BDI reduction vs. placebo + improved insulin/HOMA‐IR, hs‐CRP, glutathione.
Kazemi et al. (2019) (MDD)	DB 3‐arm RCT; *n* = 110 (81 completers); 8 weeks	*L. helveticus* + *B. longum* vs. GOS vs. placebo	Probiotic ∼10^9^ CFU/day (typical)	Probiotic reduced BDI more than placebo and GOS (*P* ≈ 0.04); modest shift in TRP–KYN markers; GOS no clear benefit.
Allen et al. (2016) (healthy)	Placebo‐controlled, within‐subject; *n* = 22; 4 weeks placebo → 4 weeks probiotic	*B. longum* 1714	1 × 10^9^ CFU/day	Lower stress‐induced cortisol and perceived stress; small but significant memory/EEG effects.
Rudzki et al. (2019) (MDD on SSRI)	DB adjunctive RCT; *n* = 79 (60 completers); 8 weeks	*L. plantarum* 299v	∼10^9^–10^1^ ^0^ CFU/day	Improved cognitive performance (APT/CVLT) + altered KYN profile; incremental antidepressant effect modest.
Miyaoka et al. (2018) (TRD inpatients)	Open‐label controlled; *n* = 40; 8 weeks	CBM588 + antidepressant vs. antidepressant only	60 mg/day	Greater HAM‐D/BDI/BAI reductions; response ∼70%, remission ∼35% (blinding limitation).
Dickerson et al. (2014) (schizophrenia)	DB RCT; *n* = 65 (58 completers); 14 weeks	LGG + Bb12	∼2 × 10^9^ CFU/day	No PANSS benefit; markedly reduced severe constipation risk (hazard ratio ≈ 0.23, *P* ≈ 0.003).
Parracho et al. (2010) (ASD)	DB crossover; *n* = 15 analysed; ∼3 week periods	*L. plantarum* WCFS1	∼4.5 × 10^1^ ^0^ CFU/day	Changed stool consistency/microbiota; behavioural effects unclear (small *n*, high dropout).
Kang et al. (2017) (ASD + GI)	Open‐label single‐arm; *n* = 18; 10 weeks + 8 weeks FU	Microbiota Transfer Therapy (multi‐step)	∼2.5 × 10^1^ ^2^ cells/day initially → ∼2.5 × 10^9^/day	∼80% GI symptom improvement + sustained ASD score improvements and higher diversity; exploratory due to open‐label, multi‐component design.

ASD, autism spectrum disorder; APT,Attention Performance Test; BAI, Beck Anxiety Inventory; BDI, Beck Depression Inventory; CFU, colony forming units; CVLT, California Verbal Learning Test; DB, double blind; FU, follow‐up; GI, gastrointestinal; GOS, galactooligosaccharide; HAM‐D, Hamilton Depression Rating Scale; HOMA‐IR, homeostatic model assessment of insulin resistance; hs‐CRP, high‐sensitivity C‐reactive protein; KYN, kynurenine; MDD, major depressive disorder; PANSS,Positive and Negative Syndrome Scale; RCT, randomized controlled trial; SSRI, selective serotonin reuptake inhibitor; TRD, treatment‐resistant depression; TRP, tryptophan.

### 5.3 Heterogeneity of outcomes

Outcomes across psychobiotic trials in mood and anxiety disorders are heterogeneous and generally modest. Evidence suggests benefits are more likely in mild to moderate depression, while effects in healthy or minimally symptomatic groups are often small or non‐significant, which may reflect ceiling effects and limited baseline HPA axis or immune dysregulation (Zhang, Chen, Zhang, et al., 2023; Zhao et al., 2025). Variability is strongly driven by strain identity, viable dose, intervention duration and sample characteristics, and may also be moderated by sex distribution and host context (Zhao et al., 2025). These patterns support the existence of responder and non‐responder subgroups shaped by baseline microbiota, diet and concomitant medications, emphasizing the need for biomarker informed trial designs (Hsiao et al., 2013).

### 5.4 Implications for clinical translation

Taken together, current evidence supports selected, strain‐specific psychobiotics (notably within *Lactobacillus*, *Bifidobacterium*, and butyrate‐related approaches) primarily as adjuncts to standard care for anxiety and depressive disorders, with effects that remain heterogeneous across trials (Gupta et al., 2026). To improve reproducibility and enable responder stratification, future studies should report strain identity and viable dose, standardize intervention duration (e.g., 8–12 weeks), and incorporate mechanistic readouts such as cytokines, cortisol, BDNF and tryptophan–kynurenine markers (Atanasova et al., 2025).

## 6 NEURODEVELOPMENTAL AND SEVERE PSYCHIATRIC CONDITIONS ARE TREATED WITH PSYCHOBIOTICS

The treatment of neurodevelopmental disorders, including ADHD and ASD, as well as severe mental illnesses like schizophrenia and bipolar disorder, is intricate. Changes in gut microbiota are increasingly being linked to their pathophysiology, primarily through impaired gut‐brain signalling, disturbed neurotransmitter balance, and early‐life immune activation (Vasiliu, 2023a). Psychobiotics may modulate these underlying mechanisms.

### 6.1 Autism spectrum disorder

Both adults and children with ASD frequently have chronic inflammation, GI symptoms, and a changed gut microbiota composition, usually with more *Clostridium* spp. And less *Bifidobacterium (* *Dash et al.*, 2022). According to Kang et al., these alterations are associated with behavioural and social impairments (Kang et al., 2017). In a mouse model of ASD (maternal immune activation), preclinical research has demonstrated that the administration of Bacteroides fragilis corrected gut permeability and restored social behaviour (Harding & Bishop, 2022). Several small‐scale clinical studies have suggested that probiotic supplements may alleviate GI symptoms, hyperactivity, and irritability. For instance, Parracho et al. discovered that *L. plantarum* WCSF1 improved social behaviour and attention in autistic children, even though the results were inconsistent and contingent upon the strain (Parracho et al., 2010).

### 6.2 Attention‐deficit/hyperactivity disorder

Executive function deficiencies and ADHD impulsivity have also been linked to the gut microbiota. The risk of developing ADHD was significantly reduced by the age of 13 through perinatal supplementation with *L. rhamnosus* GG, as indicated by a randomized trial conducted by Pärtty et al. This suggests long‐term neurodevelopmental modulation (Dash et al., 2022; Pärtty et al., 2015). Among the suggested mechanisms are modifications to tryptophan pathways, control of systemic inflammation and changes to dopamine metabolism (Novikova et al., 2024; O'Riordan et al., 2025).

### 6.3 Schizophrenia and bipolar disorder

An immunopsychiatric perspective is increasingly being used to explain severe mental illnesses such as bipolar disorder and schizophrenia, where gut dysbiosis is linked to altered blood–brain barrier permeability and systemic inflammation (Bashir & Khan, 2022). Pro‐inflammatory species are more prevalent in schizophrenia, which is frequently characterized by a reduction in microbial diversity (Vasiliu, 2023a). In a double‐blind RCT, Dickerson et al. demonstrated that a 14‐week probiotic intervention (e.g., *B. lactis* and *L. rhamnosus*) enhanced GI symptoms and reduced rehospitalization rates but had minimal impact on core psychotic symptoms (Dickerson et al., 2014). Psychobiotic trials are still in their early stages, but patients with bipolar disorder also show increased intestinal permeability and altered microbial taxa linked to mood regulation (Dash et al., 2022).

### 6.4 Limitations and interpretation

The evidence base for psychobiotics in these conditions is not as strong as it is for depression and anxiety, despite encouraging initial findings (Vasiliu, 2023a). The heterogeneity of patient populations, outcome measures and microbial baselines complicates the process of interpretation. In addition, the efficacy of treatment may be influenced by the timing of development, particularly in neurodevelopmental disorders, where early microbiota modulation may have enduring neurobehavioural effects (O'Riordan et al., 2025).

**FIGURE 3:**
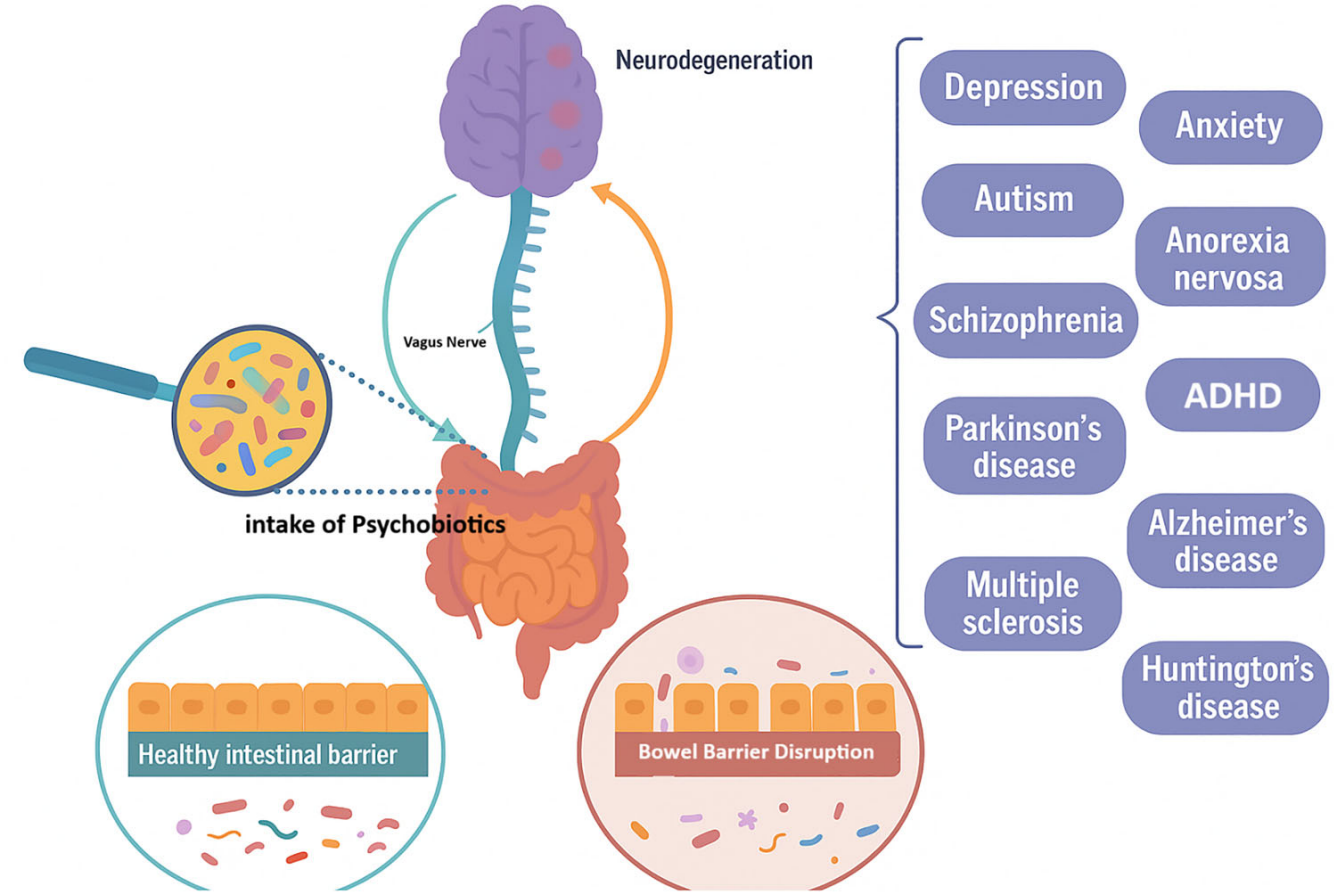
Schematic representation of the gut–brain axis highlighting the role of psychobiotics in modulating neuropsychiatric and neurodegenerative disorders. Intake of psychobiotics influences gut microbiota composition and preserves intestinal barrier integrity, which in turn communicates with the central nervous system via the vagus nerve. Disruption of the bowel barrier is implicated in the pathogenesis of various neurological and psychiatric conditions, including depression, anxiety, autism, schizophrenia, Parkinson's disease, multiple sclerosis and Alzheimer's disease.

Considering these factors, psychobiotics should currently be viewed as a promising adjunct for neurodevelopmental and severe mental illnesses, especially in patients with prominent immune or GI comorbidity (Novikova et al., 2024) (Table 3, Figure 3).

**TABLE 3:** Overview of psychobiotic applications in neurodevelopmental and severe psychiatric disorders.

Disorder	Psychobiotic strains studied	Core symptoms targeted	Proposed mechanisms	Key findings (model/trial)	Limitations	Ref.
ASD	*B. fragilis*, *L. plantarum* WCSF1	Social behaviour, GI symptoms, hyperactivity	Gut barrier integrity, neuroimmune modulation, microbial diversity	*B. fragilis* restored gut barrier and social behaviour *L. plantarum* improved attention in children	Small cohorts, inconsistent outcomes, strain‐dependent	Hsiao et al. (2013)
ADHD	*L. rhamnosus* GG	Impulsivity, executive function	Dopaminergic modulation, tryptophan pathways, inflammation control	Perinatal use reduced ADHD diagnosis by age 13	No data in treated patients; needs replication	Pärtty et al. (2015)
Schizophrenia	*B. lactis*, *L. rhamnosus*	GI distress, relapse rates	Immune regulation, BBB integrity	↓ Rehospitalization, improved GI symptoms in RCT	No effect on core psychotic features	Dickerson et al. (2014)
Bipolar disorder	Not strain‐specific	Mood instability, systemic inflammation	Gut permeability, immune markers, microbial taxa shift	Altered microbiota linked to mood regulation	No clinical trials; lacks strain precision	Painold et al. (2019)
Cross‐cutting challenges	—	GI symptoms, immune dysregulation, stress reactivity	Multimodal microbiome‐host signalling	—	Population heterogeneity, timing effects, need for biomarkers	Dinan and Cryan (2017)

### 6.5 Fecal microbiota transplantation‐based interventions in neuropsychiatric disorders

Over the past decade, fecal microbiota transplantation (FMT) has been explored as a microbiome‐based therapy for several neuropsychiatric conditions. Experimental work suggests that transferring healthy donor microbiota can reduce anxiety and depression‐like phenotypes, whereas transplantation of dysbiotic microbiota from affected individuals can induce such features in recipients (Chinna Meyyappan et al., 2020). Clinically, the most studied indications include ASD, mood and anxiety disorders, and emerging work in Alzheimer's disease (AD).

#### 6.5.1 Autism spectrum disorder

In ASD, open label studies of microbiota transfer therapy and related FMT protocols have reported marked and durable reductions in GI complaints and core autism symptoms, sometimes maintained or further improved at 2 year follow‐up, together with sustained increases in microbial diversity and enrichment of taxa such as *Bifidobacteria* and *Prevotella* (Kang et al., 2019; Zhang, Zhu, Wan, et al., 2023). Case reports and small observational cohorts consistently describe decreases in scores on scales such as ABC, CARS, and SRS after FMT. A 2023 systematic review of five ASD studies concluded that all showed significant post‐FMT symptom improvement, but emphasized that all were open label or observational and that no completed RCT was available at that time (Zhang, Zhu, Wan, et al., 2023). Early data from a larger trial suggest that placebo effects may be substantial (Maniscalco et al., 2025), which underlines the need for rigorously blinded, controlled studies before FMT can be considered beyond an experimental option for children with ASD and comorbid gut symptoms.

#### 6.5.2 Anxiety and related disorders

Anxiety‐related phenotypes have been linked to microbiota composition in both preclinical and clinical work. In animals, microbiota from highly anxious donors can transfer anxiety‐like behaviour, whereas microbiota from resilient donors can confer stress resistance (Chinna Meyyappan et al., 2020). In humans, FMT has shown anxiolytic and antidepressant effects mainly in populations with prominent GI disturbance. For example, in patients with irritable bowel syndrome and comorbid anxiety, FMT from healthy donors improved both digestive symptoms and anxiety and depression scores 1 month after treatment (Yang et al., 2023). A recent meta‐analysis in individuals with chronic insomnia also reported improvements in sleep quality and co‐occurring anxiety symptoms within 4 weeks of FMT (Zhang et al., 2025). These findings suggest that correcting gut dysbiosis may secondarily benefit mood and anxiety, but dedicated RCTs in primary anxiety disorders are still lacking.

#### 6.5.3 Depression

The antidepressant potential of FMT has been evaluated more systematically. A recent meta‐analysis of 12 RCTs including 681 participants (2019–2024) found that FMT significantly alleviated depressive symptoms compared with placebo or standard care, with effects remaining robust in sensitivity analyses (Zhang et al., 2025). One trial reported that the antidepressant benefit of FMT not only persisted but appeared to increase over time, although follow‐up durations differed between studies (Liu et al., 2024; Zhang et al., 2025). Mechanistically, FMT tends to restore microbial diversity and increase anti‐inflammatory, short chain fatty acid producing taxa, which may reduce systemic inflammation and improve neurotransmitter systems relevant to mood regulation (Liu et al., 2024). Case reports of treatment resistant depression describe marked mood improvement within weeks of adjunctive FMT after failure of conventional therapies (Liu et al., 2024; Zhang et al., 2024). Overall, the evidence suggests that microbiota transplantation can improve depression in a subset of patients (Vasiliu, 2023b), and ongoing trials are refining delivery routes and patient selection, with preliminary data hinting that colonoscopic infusion may have somewhat larger effects than oral capsules (Zhang et al., 2025).

#### 6.5.4 Alzheimer's disease and cognitive decline

In Alzheimer's disease and related cognitive disorders, FMT research is still at an early, exploratory stage. Preclinical work in transgenic AD mouse models has shown that FMT from healthy donors can improve cognition and reduce neuropathology, while shifting the recipients’ microbiota profile toward that of healthy controls (Chen et al., 2023). Clinically, case level evidence is cautiously encouraging. In one report, an AD patient who received repeated FMT showed improvement in Mini Mental State Exam score from 20 to 26 at 2 months and to 29 at 6 months post‐treatment (Sun et al., 2019). A small pilot study in patients with mild cognitive impairment found that capsule‐based FMT was safe and well tolerated, stabilized or modestly improved cognitive scores over 6 months, and produced microbial and metabolite shifts consistent with a biological effect, particularly in those with milder baseline impairment (Hazan, 2020; Sun et al., 2019). These studies involve very small samples, yet they highlight the gut–brain axis as a candidate therapeutic target in dementia. A registered feasibility study of oral FMT in AD  (Oral Fecal Microbiota Transplant Feasibility Study in Alzheimer's Disease (AMBITION), 2018) was terminated early; therefore, the clinical efficacy of FMT in larger cohorts remains to be determined. For now, FMT in Alzheimer's and related disorders should be regarded as an experimental but promising approach that requires further rigorous evaluation.

## 7 PERSONALIZED PSYCHOBIOTIC TREATMENTS: MICROBIOME‐INFORMED MENTAL HEALTH STRATEGIES

Treatment responses to psychobiotics are highly heterogeneous, influenced by host genetics, diet, medication history, environmental exposures and baseline gut microbiota (Fang et al., 2023). This variability has led to the concept of personalized microbial therapeutics tailored to an individual's clinical phenotype and microbiome profile (Rupp & Stengel, 2022). Early data suggest that baseline microbial diversity and specific taxa can predict outcomes. For example, patients with reduced *Bifidobacterium* abundance or low fecal SCFA levels often respond better to SCFA‐producing strains or prebiotics that enrich butyrate‐producing species, and individuals with MDD who respond to *L. plantarum* PS128 may show distinct increases in SCFA‐related taxa compared with non‐responders (Fang et al., 2023; Rupp & Stengel, 2022).

Near‐term clinical applications focus on simple stratification schemes. Stool metabolomic profiling could identify patients with low butyrate and high acetate to butyrate ratios who might benefit from butyrogenic formulations or synbiotics designed to restore SCFA balance (Jiang et al., 2024). Similarly, patients with elevated inflammatory markers such as C‐reactive protein, TNF‐α or IL‐6 may be better candidates for strains with documented anti‐inflammatory effects, for example *B. longum* 35624 or *L. casei* Shirota, which have reduced systemic cytokines in clinical studies (Mills et al., 2023). Such targeted strategies remain hypothesis driven and experimental, so they should be framed as exploratory, implemented with explicit consent, biomarker documentation and standardized short term follow‐up using instruments such as PHQ‐9, GAD‐7 and sleep quality indices over typical 4–12‐week intervention windows (Butler et al., 2019).

Beyond metabolomic and immune profiling, integrating host genomic information such as variants in solute carrier family 6 member 4 (*SLC6A4*) or *IL6* may further refine strain selection and response prediction, although these approaches still lack standardized validation and regulatory endorsement (Jiang et al., 2024).

## 8 CHALLENGES AND FUTURE RESEARCH DIRECTIONS

Despite encouraging preclinical and early clinical findings, several barriers still limit the translation of psychobiotics into routine mental health care. First, weak standardization across trials, including differences in strains, doses, intervention duration, delivery formats and outcome measures, makes studies hard to compare and reduces reproducibility, particularly when strain‐level reporting is incomplete (Mosquera et al., 2024). Second, regulatory ambiguity and uneven product quality remain major obstacles because many commercial products do not reliably disclose validated strain identifiers or guarantee viable dose through shelf life, which prevents direct translation of trial evidence into real‐world recommendations (Binda et al., 2020). Third, responses appear strongly host‐dependent, shaped by factors such as baseline microbiota composition, diet, sex and concomitant medications, suggesting the presence of responder and non‐responder subgroups that current trial designs do not adequately capture (Sniffen et al., 2018). Fourth, mechanistic resolution in humans is still limited because relatively few studies directly link microbial and metabolite changes to neural or synaptic outcomes using tools such as neuroimaging, electrophysiology or cerebrospinal fluid  biomarkers (Natale et al., 2021). Finally, most trials remain short, often 4–12 weeks, with limited follow‐up, so durability of benefit and longer‐term safety are not well defined (Ribeiro et al., 2025).

## 9 CLINICAL IMPLICATIONS

Current evidence supports psychobiotics as cautious, strain‐specific adjuncts in mental health care rather than stand‐alone treatments. Across trials, selected preparations may improve anxiety, depression, stress‐related outcomes and some ASD‐associated measures, particularly when GI symptoms or low‐grade inflammation are present, but effects are generally modest, heterogeneous and dependent on the exact strain, dose and intervention duration (often 4–12 weeks) (Fereshteh et al., 2020; Garzone et al., 2025; Skowron et al., 2022). In clinical practice, psychobiotics are most defensible as add‐on options for partial responders or subsyndromal presentations, and they should not replace evidence‐based treatments in severe mental illness, acute psychosis or suicidal states (Haiqa & Aslam, 2023).

A near‐term priority is improved patient and product selection. Biomarker‐ and microbiome‐informed stratification, such as targeting individuals with elevated inflammatory markers, impaired gut barrier indices or reduced SCFA‐producing taxa, remains promising but largely experimental and requires standardized monitoring and validation (Amirani et al., 2020). In parallel, the uneven quality of over‐the‐counter products, including inconsistent strain identification and uncertain viable dose across shelf life, limits direct translation from RCTs; when used, clinicians should match strain, dose and duration to published trials and preferentially select products with third‐party verification (Sharma et al., 2021).

## 10 CONCLUSION

Psychobiotics represent a promising but still emerging avenue in microbiota‐based mental health research. By acting through the MGBA, selected microbial strains and microbe derived products can modulate stress responses, immune signalling, neuroplasticity and neurotransmitter‐related pathways in ways that complement conventional psychopharmacology. Preclinical data are mechanistically robust, and early clinical trials in anxiety, depression and some neurodevelopmental conditions suggest small to moderate benefits in defined subgroups.

Translational use remains at an early stage and should be explicitly strain specific, biomarker informed where possible, and integrated with existing psychiatric treatments rather than used as a replacement. Progress will depend on rigorous strain level validation, clear reporting of dose and duration, incorporation of mechanistic biomarkers to identify likely responders, and long‐term safety data. If these conditions are met, psychobiotics may evolve from broadly marketed supplements into targeted, mechanism informed adjuncts within precision‐oriented psychiatry, particularly for patients with GI or inflammatory comorbidities.

## AUTHOR CONTRIBUTIONS

Amir Arsalan Ghahari, Mehrdad Nourizadeh, Mehrdad SalekShahabi, Shaghayegh Davari, and Saeid Mohammadzadeh Mounesyar: Conceptualization, writing – original draft, writing – review & editing, and supervision. All authors have read and approved the final version of this manuscript and agree to be accountable for all aspects of the work in ensuring that questions related to the accuracy or integrity of any part of the work are appropriately investigated and resolved. All persons designated as authors qualify for authorship, and all those who qualify for authorship are listed.

## CONFLICT OF INTEREST

None declared.

## FUNDING INFORMATION

No funding was received for this work.

## References

[eph70240-bib-0001] Accettulli, A. , Corbo, M. R. , Sinigaglia, M. , Speranza, B. , Campaniello, D. , Racioppo, A. , Altieri, C. , & Bevilacqua, A. (2022). Psycho‐microbiology, a new frontier for probiotics: An exploratory overview. Microorganisms, 10(11), 2141. 36363733 10.3390/microorganisms10112141 PMC9696884

[eph70240-bib-0002] Akkasheh, G. , Kashani‐Poor, Z. , Tajabadi‐Ebrahimi, M. , Jafari, P. , Akbari, H. , Taghizadeh, M. , Memarzadeh, M. R. , Asemi, Z. , & Esmaillzadeh, A. (2016). Clinical and metabolic response to probiotic administration in patients with major depressive disorder: A randomized, double‐blind, placebo‐controlled trial. Nutrition, 32(3), 315–320. 26706022 10.1016/j.nut.2015.09.003

[eph70240-bib-0003] Allen, A. P. , Hutch, W. , Borre, Y. E. , Kennedy, P. J. , Temko, A. , Boylan, G. , Murphy, E. , Cryan, J. F. , Dinan, T. G. , & Clarke, G. (2016). *Bifidobacterium longum* 1714 as a translational psychobiotic: Modulation of stress, electrophysiology and neurocognition in healthy volunteers. Translational Psychiatry, 6(11), e939. 27801892 10.1038/tp.2016.191 PMC5314114

[eph70240-bib-0004] Amirani, E. , Milajerdi, A. , Mirzaei, H. , Jamilian, H. , Mansournia, M. A. , Hallajzadeh, J. , & Ghaderi, A. (2020). The effects of probiotic supplementation on mental health, biomarkers of inflammation and oxidative stress in patients with psychiatric disorders: A systematic review and meta‐analysis of randomized controlled trials. Complementary Therapies in Medicine, 49, 102361. 32147043 10.1016/j.ctim.2020.102361

[eph70240-bib-0005] Andreeva, I. , Tolpygo, A. V. , Andreev, V. , Azyzov, I. , Golman, I. , Osipova, N. , Privolnev, V. , Stetsiouk, O. , & Sokolovskaya, V. (2022). Psychobiotics: A new way in psychopharmacology, or how do bacteria manage our brain? Clinical Microbiology and Antimicrobial Chemotherapy, 24(2), 108–133.

[eph70240-bib-0006] Atanasova, K. , Knödler, L.‐L. , Reindl, W. , Ebert, M. P. , & Thomann, A. K. (2025). Role of the gut microbiome in psychological symptoms associated with inflammatory bowel diseases. Seminars in Immunopathology, 47(1), 12. 39870972 10.1007/s00281-025-01036-x PMC11772462

[eph70240-bib-0007] Aziz, N. , Wal, P. , Patel, A. , & Prajapati, H. (2024). A comprehensive review on the pharmacological role of gut microbiome in neurodegenerative disorders: Potential therapeutic targets. Naunyn‐Schmiedeberg's Archives of Pharmacology, 397(10), 7307–7336. 38734839 10.1007/s00210-024-03109-4

[eph70240-bib-0008] Bashir, Y. , & Khan, A. U. (2022). The interplay between the gut‐brain axis and the microbiome: A perspective on psychiatric and neurodegenerative disorders. Frontiers in Neuroscience, 16, 1030694. 36389228 10.3389/fnins.2022.1030694 PMC9650127

[eph70240-bib-0009] Binda, S. , Hill, C. , Johansen, E. , Obis, D. , Pot, B. , Sanders, M. E. , Tremblay, A. , & Ouwehand, A. C. (2020). Criteria to QUALIFY MICROORGANISMS as “Probiotic” in foods and dietary supplements. Frontiers in Microbiology, 11, 1662. 32793153 10.3389/fmicb.2020.01662 PMC7394020

[eph70240-bib-0010] Binda, S. , Tremblay, A. , Iqbal, U. H. , Kassem, O. , Le Barz, M. , Thomas, V. , Bronner, S. , Perrot, T. , Ismail, N. , & Parker, J. A. (2024). Psychobiotics and the microbiota‐gut‐brain axis: Where do we go from here? Microorganisms, 12(4), 634. 38674579 10.3390/microorganisms12040634 PMC11052108

[eph70240-bib-0011] Bravo, J. A. , Forsythe, P. , Chew, M. V. , Escaravage, E. , Savignac, H. M. , Dinan, T. G. , Bienenstock, J. , & Cryan, J. F. (2011). Ingestion of *Lactobacillus* strain regulates emotional behavior and central GABA receptor expression in a mouse via the vagus nerve. Proceedings of the National Academy of Sciences of the United States of America, 108(38), 16050–16055. 21876150 10.1073/pnas.1102999108 PMC3179073

[eph70240-bib-0012] Butler, M. I. , Mörkl, S. , Sandhu, K. V. , Cryan, J. F. , & Dinan, T. G. (2019). The gut microbiome and mental health: What should we tell our patients?: Le microbiote Intestinal et la Santé Mentale: que Devrions‐Nous dire à nos Patients? The Canadian Journal of Psychiatry, 64(11), 747–760. 31530002 10.1177/0706743719874168 PMC6882070

[eph70240-bib-0013] Chen, G. , Shi, F. , Yin, W. , Guo, Y. , Liu, A. , Shuai, J. , & Sun, J. (2022). Gut microbiota dysbiosis: The potential mechanisms by which alcohol disrupts gut and brain functions. Frontiers in Microbiology, 13, 916765. 35966709 10.3389/fmicb.2022.916765 PMC9372561

[eph70240-bib-0014] Chen, X. , Zhang, W. , Lin, Z. , Zheng, C. , Chen, S. , Zhou, H. , & Liu, Z. (2023). Preliminary evidence for developing safe and efficient fecal microbiota transplantation as potential treatment for aged related cognitive impairments. Frontiers in Cellular and Infection Microbiology, 13, 1103189. 37113132 10.3389/fcimb.2023.1103189 PMC10127103

[eph70240-bib-0015] Chinna Meyyappan, A. , Forth, E. , Wallace, C. J. K. , & Milev, R. (2020). Effect of fecal microbiota transplant on symptoms of psychiatric disorders: A systematic review. BioMed Central Psychiatry, 20(1), 299. 32539741 10.1186/s12888-020-02654-5 PMC7294648

[eph70240-bib-0056] ClinicalTrials.gov . (2018). Oral fecal microbiota transplant feasibility study in Alzheimer's disease (AMBITION) (NCT03998423). https://clinicaltrials.gov/study/NCT03998423

[eph70240-bib-0016] Dacaya, P. , Sarapis, K. , & Moschonis, G. (2025). The role and mechanisms of probiotic supplementation on depressive symptoms: A narrative review. Current Nutrition Reports, 14(1), 53. 40153103 10.1007/s13668-025-00644-1 PMC11953144

[eph70240-bib-0017] Dash, S. , Syed, Y. A. , & Khan, M. R. (2022). Understanding the role of the gut microbiome in brain development and its association with neurodevelopmental psychiatric disorders. Frontiers in Cell and Developmental Biology, 10, 880544. 35493075 10.3389/fcell.2022.880544 PMC9048050

[eph70240-bib-0018] Del Toro‐Barbosa, M. , Hurtado‐Romero, A. , Garcia‐Amezquita, L. , & García‐Cayuela, T. (2020). Psychobiotics: Mechanisms of action, evaluation methods and effectiveness in applications with food products. Nutrients, 12(12), 3896. 33352789 10.3390/nu12123896 PMC7767237

[eph70240-bib-0019] Dickerson, F. B. , Stallings, C. , Origoni, A. , Katsafanas, E. , Savage, C. L. , Schweinfurth, L. A. , Goga, J. , Khushalani, S. , & Yolken, R. H. (2014). Effect of probiotic supplementation on schizophrenia symptoms and association with gastrointestinal functioning: A randomized, placebo‐controlled trial. *Primary Care Companion for CNS Disorders*, 16(1), PCC.13m01579. 10.4088/PCC.13m01579 PMC4048142 24940526

[eph70240-bib-0020] Dinan, T. G. , & Cryan, J. F. (2017). Gut instincts: Microbiota as a key regulator of brain development, ageing and neurodegeneration. The Journal of Physiology, 595(2), 489–503. 27641441 10.1113/JP273106 PMC5233671

[eph70240-bib-0021] Dinan, T. G. , Stanton, C. , & Cryan, J. F. (2013). Psychobiotics: A novel class of psychotropic. Biological Psychiatry, 74(10), 720–726. 23759244 10.1016/j.biopsych.2013.05.001

[eph70240-bib-0022] Fang, H. , Yao, T. , Li, W. , Pan, N. , Xu, H. , Zhao, Q. , Su, Y. , Xiong, K. , & Wang, J. (2023). Efficacy and safety of fecal microbiota transplantation for chronic insomnia in adults: A real world study. Frontiers in Microbiology, 14, 1299816. 38088972 10.3389/fmicb.2023.1299816 PMC10712199

[eph70240-bib-0023] Fereshteh, A. , Hadi, P. , Aydin, T. , & Aziz, H. (2020). The effects of probiotics and prebiotics on mental disorders: A review on depression, anxiety, Alzheimer, and autism spectrum disorders. Current Pharmaceutical Biotechnology, 21(7), 555–565. 31914909 10.2174/1389201021666200107113812

[eph70240-bib-0024] Garzone, S. , Charitos, I. , Mandorino, M. , Maggiore, M. , Capozzi, L. , Cakani, B. , Lopes, G. C. D. , Bocchio‐Chiavetto, L. , & Colella, M. (2025). Can we modulate our second brain and its metabolites to change our mood? A systematic review on efficacy, mechanisms, and future directions of “Psychobiotics”. International Journal of Molecular Sciences, 26(5), 1972. 40076598 10.3390/ijms26051972 PMC11899754

[eph70240-bib-0025] Generoso, J. , Giridharan, V. , Lee, J. , Macedo, D. , & Barichello, T. (2020). The role of the microbiota‐gut‐brain axis in neuropsychiatric disorders. Brazilian Journal of Psychiatry, 43(3), 293–305. 10.1590/1516-4446-2020-0987 PMC8136391 32667590

[eph70240-bib-0026] GBD 2019 Mental Disorders Collaborators . (2022). Global, regional, and national burden of 12 mental disorders in 204 countries and territories, 1990‐2019: A systematic analysis for the Global Burden of Disease Study 2019. Lancet Psychiatry, 9(2), 137–150. 35026139 10.1016/S2215-0366(21)00395-3 PMC8776563

[eph70240-bib-0027] Gupta, M. K. , Chauhan, K. , Bhardwaj, S. , & Srivastava, R. (2026). Innovative interventions: Postbiotics and psychobiotics in neurodegenerative disease treatment. Probiotics and Antimicrobial Proteins, 18, 2818–2837. 40576748 10.1007/s12602-025-10632-0

[eph70240-bib-0028] Haiqa, Z. , & Aslam, U. (2023). Should probiotics be administered as an adjunctive treatment along with antidepressants for major depressive disorder? Journal of the Pakistan Medical Association, 74(1), 197–197. 10.47391/JPMA.7782 38219207

[eph70240-bib-0029] Harding, S. L. , & Bishop, J. (2022). The gut microbiome, mental health, and cognitive and neurodevelopmental disorders: A scoping review. The Journal for Nurse Practitioners, 18(7), 719–725.

[eph70240-bib-0030] Hazan, S. (2020). Rapid improvement in Alzheimer's disease symptoms following fecal microbiota transplantation: A case report. Journal of International Medical Research, 48(6), 0300060520925930. 32600151 10.1177/0300060520925930 PMC7328362

[eph70240-bib-0031] Hsiao, E. Y. , McBride, S. W. , Hsien, S. , Sharon, G. , Hyde, E. R. , McCue, T. , Codelli, J. A. , Chow, J. , Reisman, S. E. , Petrosino, J. F. , Patterson, P. H. , & Mazmanian, S. K. (2013). Microbiota modulate behavioral and physiological abnormalities associated with neurodevelopmental disorders. Cell, 155(7), 1451–1463. 24315484 10.1016/j.cell.2013.11.024 PMC3897394

[eph70240-bib-0032] Hunjan, G. , Shah, S. S. , Kosey, S. , & Aran, K. R. (2025). Gut microbiota and the tryptophan‐kynurenine pathway in anxiety: New insights and treatment strategies. Journal of Neural Transmission, 132(7), 943–977. 40369368 10.1007/s00702-025-02938-8

[eph70240-bib-0033] Jia, L. , Xiao, L. , Fu, Y. , Shao, Z. , Jing, Z. , Yuan, J. , Xie, Y. , Guo, J. , Wang, Y. , & Geng, W. (2024). Neuroprotective effects of probiotics on anxiety‐ and depression‐like disorders in stressed mice by modulating tryptophan metabolism and the gut microbiota. Food & function, 15(6), 2895–2905. 38404190 10.1039/d3fo03897a

[eph70240-bib-0034] Jiang, Y. , Qu, Y. , Shi, L. , Ou, M. , Du, Z. , Zhou, Z. , Zhou, H. , & Zhu, H. (2024). The role of gut microbiota and metabolomic pathways in modulating the efficacy of SSRIs for major depressive disorder. Translational Psychiatry, 14(1), 493. 39695082 10.1038/s41398-024-03208-z PMC11655517

[eph70240-bib-0035] Kang, D.‐W. , Adams, J. B. , Coleman, D. M. , Pollard, E. L. , Maldonado, J. , McDonough‐Means, S. , Caporaso, J. G. , & Krajmalnik‐Brown, R. (2019). Long‐term benefit of Microbiota Transfer Therapy on autism symptoms and gut microbiota. Scientific Reports, 9(1), 5821. 30967657 10.1038/s41598-019-42183-0 PMC6456593

[eph70240-bib-0036] Kang, D. W. , Adams, J. B. , Gregory, A. C. , Borody, T. , Chittick, L. , Fasano, A. , Khoruts, A. , Geis, E. , Maldonado, J. , McDonough‐Means, S. , Pollard, E. L. , Roux, S. , Sadowsky, M. J. , Lipson, K. S. , Sullivan, M. B. , Caporaso, J. G. , & Krajmalnik‐Brown, R. (2017). Microbiota Transfer Therapy alters gut ecosystem and improves gastrointestinal and autism symptoms: An open‐label study. Microbiome, 5(1), 10. 28122648 10.1186/s40168-016-0225-7 PMC5264285

[eph70240-bib-0037] Kar, F. , Hacioglu, C. , Kar, E. , Donmez, D. B. , & Kanbak, G. (2022). Probiotics ameliorates LPS induced neuroinflammation injury on Aβ 1‐42, APP, γ‐β secretase and BDNF levels in maternal gut microbiota and fetal neurodevelopment processes. Metabolic Brain Disease, 37(5), 1387–1399. 35312928 10.1007/s11011-022-00964-z

[eph70240-bib-0038] Kazemi, A. , Noorbala, A. A. , Azam, K. , Eskandari, M. H. , & Djafarian, K. (2019). Effect of probiotic and prebiotic vs placebo on psychological outcomes in patients with major depressive disorder: A randomized clinical trial. Clinical Nutrition, 38(2), 522–528. 29731182 10.1016/j.clnu.2018.04.010

[eph70240-bib-0039] Kim, I. B. , Park, S.‐C. , & Kim, Y.‐K. (2023). Microbiota‐gut‐brain axis in major depression: A new therapeutic approach. In Y.‐K. Kim (Ed.), Neuroinflammation, Gut‐Brain Axis and Immunity in Neuropsychiatric Disorders (pp. 209–224). Springer Nature Singapore. 10.1007/978-981-19-7376-5_10 36949312

[eph70240-bib-0040] Lachmansingh, D. A. , Lavelle, A. , Cryan, J. F. , & Clarke, G. (2024). Microbiota‐gut‐brain axis and antidepressant treatment. In M. Browning , P. J. Cowen , & T. Sharp (Eds.), Emerging Neurobiology of Antidepressant Treatments (pp. 175–216). Springer International Publishing. 10.1007/7854_2023_449 37962812

[eph70240-bib-0041] Lai, H.‐Y. , & Shen, T. (2023). Gut microbiota, alzheimer and psychiatric diseases: Unveiling the relationships and treatment options. In F. Marotta (Ed.), Gut Microbiota in Aging and Chronic Diseases (pp. 279–333). Springer International Publishing.

[eph70240-bib-0042] Li, J. , Li, Y. , Zhao, J. , Li, L. , Wang, Y. , Chen, F. , Li, Y. , Cheng, R. , He, F. , Ze, X. , & Shen, X. (2024). Effects of *Bifidobacterium breve* 207‐1 on regulating lifestyle behaviors and mental wellness in healthy adults based on the microbiome‐gut‐brain axis: A randomized, double‐blind, placebo‐controlled trial. European Journal of Nutrition, 63(7), 2567–2585. 38869657 10.1007/s00394-024-03447-2

[eph70240-bib-0043] Liu, P. , Liu, Z. , Wang, J. , Wang, J. , Gao, M. , Zhang, Y. , Yang, C. , Zhang, A. , Li, G. , Li, X. , Liu, S. , Liu, L. , Sun, N. , & Zhang, K. (2024). Immunoregulatory role of the gut microbiota in inflammatory depression. Nature Communications, 15(1), 3003. 10.1038/s41467-024-47273-w PMC11001948 38589368

[eph70240-bib-0044] Magalhães‐Guedes, K. , Nascimento, A. S. M. D. , Anunciação, T. , & Soares, S. (2020). Psychobiotics in daily food against psychiatric disorders. Australian Journal of French Studies, 14, 161–166.

[eph70240-bib-0045] Maniscalco, I. , Bartochowski, P. , Priori, V. , Iancau, S. P. , De Francesco, M. , Innamorati, M. , Jagodzinska, N. , Giupponi, G. , Masucci, L. , Conca, A. , & Mroczek, M. (2025). The effects of fecal microbial transplantation on the symptoms in autism spectrum disorder, gut microbiota and metabolites: A scoping review. Microorganisms, 13(6), 1290. 40572178 10.3390/microorganisms13061290 PMC12195398

[eph70240-bib-0046] Mazziotta, C. , Tognon, M. , Martini, F. , Torreggiani, E. , & Rotondo, J. C. (2023). Probiotics mechanism of action on immune cells and beneficial effects on human health. Cells, 12(1), 184. 36611977 10.3390/cells12010184 PMC9818925

[eph70240-bib-0047] Mills, S. , Yang, B. , Smith, G. J. , Stanton, C. , & Ross, R. P. (2023). Efficacy of *Bifidobacterium longum* alone or in multi‐strain probiotic formulations during early life and beyond. Gut Microbes, 15(1), 2186098. 36896934 10.1080/19490976.2023.2186098 PMC10012958

[eph70240-bib-0048] Miyaoka, T. , Kanayama, M. , Wake, R. , Hashioka, S. , Hayashida, M. , Nagahama, M. , Okazaki, S. , Yamashita, S. , Miura, S. , Miki, H. , Matsuda, H. , Koike, M. , Izuhara, M. , Araki, T. , Tsuchie, K. , Azis, I. A. , Arauchi, R. , Abdullah, R. A. , Oh‐Nishi, A. , & Horiguchi, J. (2018). *Clostridium butyricum* MIYAIRI 588 as adjunctive therapy for treatment‐resistant major depressive disorder: A prospective open‐label trial. Clinical Neuropharmacology, 41(5), 151–155. 30234616 10.1097/WNF.0000000000000299

[eph70240-bib-0049] Moerkl, S. , Butler, M. , Holl, A. , Cryan, J. , & Dinan, T. (2020). Probiotics and the microbiota‐gut‐brain axis: Focus on psychiatry. Current Nutrition Reports, 9(3), 171–182. 32406013 10.1007/s13668-020-00313-5 PMC7398953

[eph70240-bib-0050] Mohammadi, G. , Dargahi, L. , Peymani, A. , Mirzanejad, Y. , Alizadeh, S. A. , Naserpour, T. , & Nassiri‐Asl, M. (2019). The effects of probiotic formulation pretreatment (*Lactobacillus helveticus* R0052 and *Bifidobacterium longum* R0175) on a lipopolysaccharide rat model. Journal of the American College of Nutrition, 38(3), 209–217. 30307792 10.1080/07315724.2018.1487346

[eph70240-bib-0051] Mosquera, F. E. C. , Liscano, Y. , & Martinez, S. L. (2024). Effectiveness of psychobiotics in the treatment of psychiatric and cognitive disorders: A systematic review of randomized clinical trials. Nutrients, 16(9), 1352. 38732599 10.3390/nu16091352 PMC11085935

[eph70240-bib-0052] Natale, N. R. , Kent, M. , Fox, N. , Vavra, D. , & Lambert, K. (2021). Neurobiological effects of a probiotic‐supplemented diet in chronically stressed male Long‐Evans rats: Evidence of enhanced resilience. International Brain Research Organization, Neuroscience Reports, 11, 207–215. 10.1016/j.ibneur.2021.10.004 PMC8607205 34849506

[eph70240-bib-0053] Neska, A. , Kędzierska, E. , & Gibuła‐Tarłowska, E. (2024). The impact of gut microbiota on mental health. Current Issues in Pharmacy and Medical Sciences, 37(4), 226–231.

[eph70240-bib-0054] Novikova, V. A. , Bondarenko, K. D. , Sazonov, A. E. , & Rozanov, A. S. (2024). The effect of probiotic lactic acid bacteria on the symptoms of mental disorders. Nanobiotechnology Reports, 19(5), 645–666.

[eph70240-bib-0055] O'Riordan, K. J. , Aburto, M. R. , Nagpal, J. , Clarke, G. , & Cryan, J. F. (2025). Microbiome: A key regulator of body‐brain interactions. In D. Tropea & E. Giacometti (Eds.), Brain‐Body Connections: Bidirectional Communication Between the Brain and Body Systems (pp. 139–203). Springer Nature Switzerland. 10.1007/978-3-031-89525-8_6 40442386

[eph70240-bib-0057] Oroojzadeh, P. , Bostanabad, S. Y. , & Lotfi, H. (2022). Psychobiotics: The influence of gut microbiota on the gut‐brain axis in neurological disorders. Journal of Molecular Neuroscience, 72(9), 1952–1964. 35849305 10.1007/s12031-022-02053-3 PMC9289355

[eph70240-bib-0058] Painold, A. , Mörkl, S. , Kashofer, K. , Halwachs, B. , Dalkner, N. , Bengesser, S. , Birner, A. , Fellendorf, F. , Platzer, M. , Queissner, R. , Schütze, G. , Schwarz, M. J. , Moll, N. , Holzer, P. , Holl, A. K. , Kapfhammer, H.‐P. , Gorkiewicz, G. , & Reininghaus, E. Z. (2019). A step ahead: Exploring the gut microbiota in inpatients with bipolar disorder during a depressive episode. Bipolar Disorders, 21(1), 40–49. 30051546 10.1111/bdi.12682 PMC6585963

[eph70240-bib-0059] Parracho, H. , Gibson, G. R. , Knott, F. , Bosscher, D. , Kleerebezem, M. , & McCartney, A. (2010). A double‐blind, placebo‐controlled, crossover‐designed probiotic feeding study in children diagnosed with autistic spectrum disorders. International Journal of Probiotics and Prebiotics, 5, 69–74.

[eph70240-bib-0060] Pärtty, A. , Kalliomäki, M. , Wacklin, P. , Salminen, S. , & Isolauri, E. (2015). A possible link between early probiotic intervention and the risk of neuropsychiatric disorders later in childhood: A randomized trial. Pediatric Research, 77(6), 823–828. 25760553 10.1038/pr.2015.51

[eph70240-bib-0061] Ramadan, Y. N. , Alqifari, S. F. , Alshehri, K. , Alhowiti, A. , Mirghani, H. , Alrasheed, T. , Aljohani, F. , Alghamdi, A. , & Hetta, H. F. (2025). Microbiome gut‐brain‐axis: Impact on brain development and mental health. Molecular Neurobiology, 62(8), 10813–10833. 40234288 10.1007/s12035-025-04846-0 PMC12289773

[eph70240-bib-0062] Ribeiro, G. , Schellekens, H. , Cuesta‐Marti, C. , Maneschy, I. , Ismael, S. , Cuevas‐Sierra, A. , Martínez, J. A. , Silvestre, M. P. , Marques, C. , Moreira‐Rosário, A. , Faria, A. , Moreno, L. A. , & Calhau, C. (2025). A menu for microbes: Unraveling appetite regulation and weight dynamics through the microbiota‐brain connection across the lifespan. American Journal of Physiology. Gastrointestinal and Liver Physiology, 328(3), G206–G228. 39811913 10.1152/ajpgi.00227.2024

[eph70240-bib-0063] Roy, S. , Bhowmick, P. , Banerjee, S. , Choudhury, L. , & Mukherjee, A. (2024). Chapter 12 – Neuropsychiatric applications of psychobiotics. In R. Pratap Singh , G. Manchanda , S. Sarsan , A. Kumar , & H. Panosyan (Eds.), Microbial Essentialism (pp. 301–315). Academic Press.

[eph70240-bib-0064] Rudzki, L. , Ostrowska, L. , Pawlak, D. , Małus, A. , Pawlak, K. , Waszkiewicz, N. , & Szulc, A. (2019). Probiotic *Lactobacillus plantarum* 299v decreases kynurenine concentration and improves cognitive functions in patients with major depression: A double‐blind, randomized, placebo controlled study. Psychoneuroendocrinology, 100, 213–222. 30388595 10.1016/j.psyneuen.2018.10.010

[eph70240-bib-0065] Rupp, S. K. , & Stengel, A. (2022). bi‐directionality of the microbiota‐gut‐brain axis in patients with functional dyspepsia: Relevance of psychotherapy and probiotics. Frontiers in Neuroscience, 16, 844564. 35295092 10.3389/fnins.2022.844564 PMC8919856

[eph70240-bib-0066] Samtiya, M. , Dhewa, T. , & Puniya, A. K. (2022). Probiotic mechanism to modulate the gut‐brain axis (GBA). In R. Z. Sayyed & M. Khan (Eds.), Microbiome‐Gut‐Brain Axis: Implications on Health (pp. 237–259). Springer Nature Singapore.

[eph70240-bib-0067] Sharma, R. , Gupta, D. , Mehrotra, R. , & Mago, P. (2021). Psychobiotics: The next‐generation probiotics for the brain. Current Microbiology, 78(2), 449–463. 33394083 10.1007/s00284-020-02289-5

[eph70240-bib-0068] Skowron, K. , Budzyńska, A. , Wiktorczyk‐Kapischke, N. , Chomacka, K. , Grudlewska‐Buda, K. , Wilk, M. , Wałecka‐Zacharska, E. , Andrzejewska, M. , & Gospodarek‐Komkowska, E. (2022). The role of psychobiotics in supporting the treatment of disturbances in the functioning of the nervous system—A systematic review. International Journal of Molecular Sciences, 23(14), 7820. 35887166 10.3390/ijms23147820 PMC9319704

[eph70240-bib-0069] Sniffen, J. C. , McFarland, L. V. , Evans, C. T. , & Goldstein, E. J. C. (2018). Choosing an appropriate probiotic product for your patient: An evidence‐based practical guide. PLoS ONE, 13(12), e0209205. 30586435 10.1371/journal.pone.0209205 PMC6306248

[eph70240-bib-0070] Sun, J. , Xu, J. , Ling, Y. , Wang, F. , Gong, T. , Yang, C. , Ye, S. , Ye, K. , Wei, D. , & Song, Z. (2019). Fecal microbiota transplantation alleviated Alzheimer's disease‐like pathogenesis in APP/PS1 transgenic mice. Translational Psychiatry, 9(1), 189. 31383855 10.1038/s41398-019-0525-3 PMC6683152

[eph70240-bib-0071] Takada, M. , Nishida, K. , Kataoka‐Kato, A. , Gondo, Y. , Ishikawa, H. , Suda, K. , Kawai, M. , Hoshi, R. , Watanabe, O. , Igarashi, T. , Kuwano, Y. , Miyazaki, K. , & Rokutan, K. (2016). Probiotic *Lactobacillus casei* strain Shirota relieves stress‐associated symptoms by modulating the gut‐brain interaction in human and animal models. Neurogastroenterol Motil, 28(7), 1027–1036. 26896291 10.1111/nmo.12804

[eph70240-bib-0072] Tiwari, S. , & Paramanik, V. (2025). Role of probiotics in depression: Connecting dots of gut‐brain‐axis through hypothalamic‐pituitary adrenal axis and tryptophan/kynurenic pathway involving indoleamine‐2,3‐dioxygenase. Molecular Neurobiology, 62(6), 7230–7241. 39875781 10.1007/s12035-025-04708-9

[eph70240-bib-0073] Vasiliu, O. (2023a). The current state of research for psychobiotics use in the management of psychiatric disorders–A systematic literature review. Frontiers in Psychiatry, 14, 1074736. 36911130 10.3389/fpsyt.2023.1074736 PMC9996157

[eph70240-bib-0074] Vasiliu, O. (2023b). Is fecal microbiota transplantation a useful therapeutic intervention for psychiatric disorders? A narrative review of clinical and preclinical evidence. Current Medical Research and Opinion, 39(1), 161–177. 36094098 10.1080/03007995.2022.2124071

[eph70240-bib-0075] Wadan, A.‐H. S. , El‐Aziz, M. K. A. , & Ellakwa, D. E.‐S. (2025). The microbiota‐gut‐brain‐axis theory: Role of gut microbiota modulators (GMMs) in gastrointestinal, neurological, and mental health disorders. Naunyn‐Schmiedeberg's Archives of Pharmacology, 398(10), 13397–13426. 40323507 10.1007/s00210-025-04155-2 PMC12511254

[eph70240-bib-0076] Wang, H. , Braun, C. , Murphy, E. F. , & Enck, P. (2019). *Bifidobacterium longum* 1714™ strain modulates brain activity of healthy volunteers during social stress. The American Journal of Gastroenterology, 114(7), 1152–1162. 30998517 10.14309/ajg.0000000000000203 PMC6615936

[eph70240-bib-0077] Wu, W. , Li, S. , & Ye, Z. (2025). Targeting the gut microbiota‐inflammation‐brain axis as a potential therapeutic strategy for psychiatric disorders: A Mendelian randomization analysis. Journal of Affective Disorders, 374, 150–159. 39809351 10.1016/j.jad.2025.01.050

[eph70240-bib-0078] Yadav, M. , Kumar, A. , & Shandil, A. (2022). Therapeutic mechanisms of gut microbiota and probiotics in the management of mental disorders. In P. K. Deol & S. K. Sandhu (Eds.), Probiotic Research in Therapeutics: Volume 4: Probiotics in Neurodegenerative Disorders (pp. 53–67). Springer Nature Singapore.

[eph70240-bib-0079] Yamawaki, Y. , Yoshioka, N. , Nozaki, K. , Ito, H. , Oda, K. , Harada, K. , Shirawachi, S. , Asano, S. , Aizawa, H. , Yamawaki, S. , Kanematsu, T. , & Akagi, H. (2018). Sodium butyrate abolishes lipopolysaccharide‐induced depression‐like behaviors and hippocampal microglial activation in mice. Brain Research, 1680, 13–38. 29229502 10.1016/j.brainres.2017.12.004

[eph70240-bib-0080] Yang, C. , Hu, T. , Xue, X. , Su, X. , Zhang, X. , Fan, Y. , Shen, X. , & Dong, X. (2023). Multi‐omics analysis of fecal microbiota transplantation's impact on functional constipation and comorbid depression and anxiety. BioMed Central Microbiology, 23(1), 389. 38057705 10.1186/s12866-023-03123-1 PMC10701952

[eph70240-bib-0081] Yousuf, B. , Mottawea, W. , Esmail, G. A. , Nazemof, N. , Bouhlel, N. E. , Njoku, E. , Li, Y. , Zhang, X. , Minic, Z. , & Hammami, R. (2025). Multi‐omics unveils strain‐specific neuroactive metabolite production linked to inflammation modulation by Bacteroides and their extracellular vesicles. Current Research in Microbial Sciences, 8, 100358. 40027450 10.1016/j.crmicr.2025.100358 PMC11868947

[eph70240-bib-0082] Zakharova, I. , Dmitrieva, D. , Berezhnaya, I. , Serikova, L. , Sugyan, N. , & Gostyukhina, A. (2022). Clinical effects of probiotics on the functioning of the gut‐brain axis in children. Meditsinskiy sovet = Medical Council, 12, 152–159.

[eph70240-bib-0083] Zhang, J. , Zhu, G. , Wan, L. , Liang, Y. , Liu, X. , Yan, H. , Zhang, B. , & Yang, G. (2023). Effect of fecal microbiota transplantation in children with autism spectrum disorder: A systematic review. Frontiers in Psychiatry, 14, 1123658. 36937721 10.3389/fpsyt.2023.1123658 PMC10017995

[eph70240-bib-0084] Zhang, Q. , Bi, Y. , Zhang, B. , Jiang, Q. , Mou, C. K. , Lei, L. , Deng, Y. , Li, Y. , Yu, J. , Liu, W. , & Zhao, J. (2024). Current landscape of fecal microbiota transplantation in treating depression. Frontiers in Immunology, 15, 1416961. 38983862 10.3389/fimmu.2024.1416961 PMC11231080

[eph70240-bib-0085] Zhang, Q. , Chen, B. , Zhang, J. , Dong, J. , Ma, J. , Zhang, Y. , Jin, K. , & Lu, J. (2023). Effect of prebiotics, probiotics, synbiotics on depression: Results from a meta‐analysis. BioMed Central Psychiatry, 23(1), 477. 37386630 10.1186/s12888-023-04963-x PMC10308754

[eph70240-bib-0086] Zhang, X. , Li, Y. , Guo, Y. , Sun, J. , & Yang, Y. (2025). Clinical efficacy of fecal microbiota transplantation in alleviating depressive symptoms: A meta‐analysis of randomized trials. Frontiers in Psychiatry, 16, 1656969. 41122746 10.3389/fpsyt.2025.1656969 PMC12536323

[eph70240-bib-0087] Zhao, S. , Liang, S. , Tao, J. , Peng, Y. , Chen, S. , Wai, H. K. F. , Chung, F. Y. , Sin, Z. Y. , Wong, M. K. L. , Haqq, A. M. , Chang, W. C. , Ni, M. Y. , Chan, F. K. L. , Ng, S. C. , & Tun, H. M. (2025). Probiotics for adults with major depressive disorder compared with antidepressants: A systematic review and network meta‐analysis. Nutrition Reviews, 83(1), 72–82. 10.1093/nutrit/nuad171 38219239

[eph70240-bib-0088] Zielińska, D. , Karbowiak, M. , & Brzezicka, A. (2022). The role of psychobiotics to ensure mental health during the COVID‐19 pandemic—a current state of knowledge. International Journal of Environmental Research and Public Health, 19(17), 11022. 36078738 10.3390/ijerph191711022 PMC9518511

[eph70240-bib-0089] Zubareva, O. E. , & Melik‐Kasumov, T. B. (2021). The gut–brain axis and peroxisome proliferator‐activated receptors in the regulation of epileptogenesis. Journal of Evolutionary Biochemistry and Physiology, 57(4), 743–760.

